# From the Old, the Best: Parathyroidectomy in the Management of Soft-Tissue and Vascular Calcification in Patients with Chronic Renal Disease

**DOI:** 10.1155/2021/9985308

**Published:** 2021-11-09

**Authors:** P. Mariel Hernandez, B. Daniel Enos, T. Gonzalo Labarca, A. Guillermo Vanderstelt

**Affiliations:** ^1^Department of Nephrology, Healthcare Complex Dr. Victor Rios Ruiz (CAVRR), Los Angeles, Biobio Province, Chile; ^2^Internal Medicine Service, CAVRR, Los Angeles, Biobio Province, Chile; ^3^Head and Neck Surgeon, Surgery Service, CAVRR, Los Angeles, Biobio Province, Chile

## Abstract

*Introduction*. Bone mineral disease in patients with chronic kidney disease (CKD-MBD) is a clinical syndrome involving bone, biochemical changes, and extraosseous calcification. These complications increase morbidity and mortality. Prevalence reports are rare. *Case Report*. This case shows a young woman on peritoneal dialysis (PD) for 10 years with severe secondary hyperparathyroidism and soft-tissue calcifications in the hands, pelvis, and right knee, as well as severe vascular calcification, managed with calcimimetics without success. We decided to perform subtotal parathyroidectomy (STPTX). Three months after surgery, she had satisfactory evolution, despite notable hungry bone disease, without bone pain or functional limitation and almost no calcifications. *Discussion*. The benefit of hemodialysis has been shown with better volume management and improvement of calcium/phosphate products. STPTX allowed biochemical control and calcification improvement, with an evident better quality of life for our patient. Therapeutic alternatives need to be tailored to the patient's characteristics in the calcimimetics era.

## 1. Introduction

Soft-tissue calcifications are frequent features of bone mineral disease in patients with chronic kidney disease (CKD-MBD), increasing mortality risk [1, 2]. Tumoral calcifications were first described in 1943 and were characterized by one or more periarticular calcifications. Calciphylaxis has been reported to occur in 1 to 4.5% of dialysis patients, including 1.6% prevalence in peritoneal dialysis (PD) [3].

Vascular calcification is a continuous biological process of calcium and phosphate metabolic dysregulation, featuring calcium deposits elsewhere (either in high- or low-turnover bone disease). These include active cells allowing mineral deposits on vessels wall, mimicking bone formation, as a dynamic process over vascular smooth muscle cells (VSMCs). As a result, it makes osteogenic differentiation and synthesis of several abnormal proteins, such as collagen type 1, osteocalcin, osteopontin, alkaline phosphatase, RUNX2, and BMP‐2, leading to matrix mineralization [4]. Serum phosphate levels are positively associated with nutritional biomarkers and PD adequacy, indicating a high daily phosphate intake and PD inadequacy may accelerate extraosseous calcification progression [4, 5].

The diagnosis of calcification begins with a thoroughly anamnesis and complete physical examination to identify deformities in soft tissue and bone structures, functional capacity, and skin lesions. The components supporting the diagnosis of CKD‐MBD include the detection of extraosseous calcification involving arterial, valvular, and myocardial calcification. Thus, the 2017 Kidney Disease: Improving Global Outcomes (KDIGO) guidelines recommend widely available techniques, such as lateral abdominal X‐ray and echocardiography which may be a good support for better diagnosis [6].

Treatment recommendations are based on weak evidence, and therapeutic failures are common. The wide range of suggested treatment options are a good proof of uncertain efficacy and include sodium thiosulfate, bisphosphonates, calcimimetics, emergency parathyroidectomy (PTX), exchange of plasma, and dialysis treatment optimization [2, 7, 8].

The present case describes the development, treatment, and outcome of massive vascular and soft-tissue calcification in a patient receiving PD before and after STPTX.

## 2. Case Report

A 23-year-old woman, with probable congenital noncorrected ureter vesical reflux, has been under PD treatment from ten years ago (2010) after a kidney transplantation failure. She had severe secondary hyperparathyroidism, with important bone and articular pain, specifically in the knees and hands. The physical examination revealed two masses, one in the left hand and the other in the right knee for over two years without pathological fractures. Those two masses caused her severe movement limitation, even preventing her to wear trousers.

She was initially treated at another health center, with no proper dialysis prescription compliance. She was transferred to our hospital with clinical evidence of this bad compliance, using oral calcitriol at different doses, cinacalcet 60 mg po/day (recently started), sevelamer 7200 mg po/day, and erythropoietin (EPO) 8000 U/weekly. Paricalcitol and etelcalcetide were not available. She received calcium-based P binders for a long time and afterwards switched to sevelamer last year because it was not available previously at the health center.

Laboratory investigations showed intact parathormone (iPTH) 1936 pg/ml (by chemiluminescent microparticle immunoassay (CMIA), normal values (NV): 15–65 pg/ml), calcium (Ca) 7.6 mg/dl (NV: 8.4–10.2 mg/dl), phosphate (P) 9.1 mg/dl (NV: 2.3–4.7 mg/dL), and alkaline phosphatase (AP) 277 U/L (by the PNP kinetic method, NV: 40–150 U/L).

**Figure 1:**
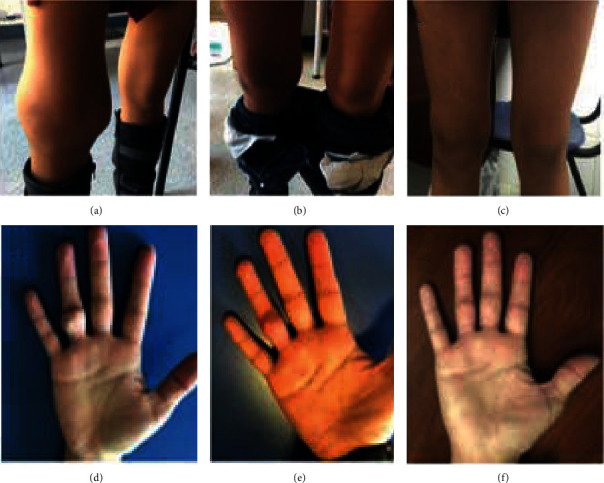
Soft-tissue calcification of the knee before surgery and 1 and 3 months after surgery (a, b, and c, respectively) and of the 4th right hand finger before surgery and 1 and 3 months after surgery (d, e, and f, respectively).

**Figure 2:**
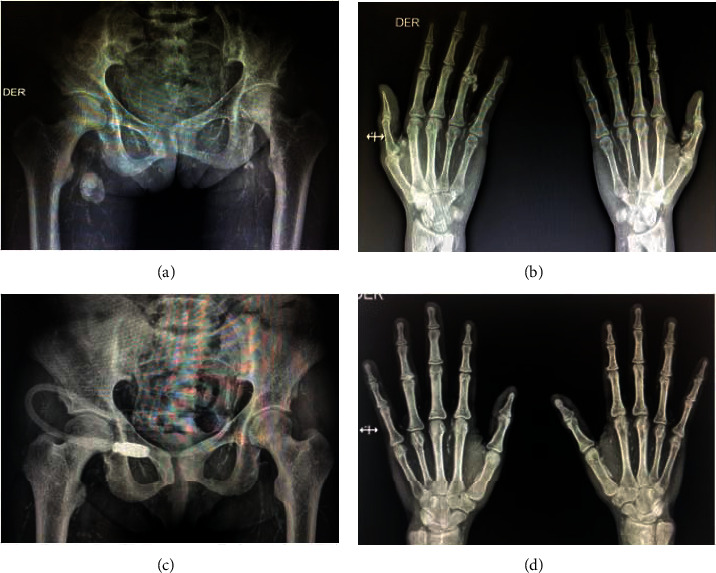
Soft-tissue and vascular calcification in Rx of the pelvis and hands before surgery (a, b) with Adragao score 8/8 and 3 months after surgery (c, d) with Adragao score 6/8.

Physical examination and radiographies showed many calcifications in her soft tissues and hands, with an Adragao score [9] of 8/8 at baseline (Figures 1 and 2). Parathyroid ultrasound showed two hyperplastic inferior glands, sized 11 × 5.4 mm in the right gland and 8.7 × 5.9 mm in the left gland. Echocardiography found moderate-to-severe pericardial effusion in the context of an insufficient dialysis dose. Calcitriol was withdrawn due to severe hyperphosphatemia. She was switched to daily hemodialysis (HD) with a low-calcium bath (2.5 mEq/L) for pericardial effusion management and achieved better control after 10 days in preparation for surgical resolution according to indications referenced by Lau et al. [10], staying in this dialysis modality after surgery.

A subtotal parathyroidectomy was performed, leaving a 30% remnant of the right inferior gland, with iPTH decreased to 205 pg/ml in the intraoperatory sample. Her postoperative course was complicated with severe hungry bone disease (HBD), requiring a high dose of calcium, first intravenous, then oral, and oral substituted vitamin *D* supplementation, following the protocol of Sampaio et al. [11] She was discharged on day 9, with full oral medication (calcitriol 1.5 mg po/day and oral calcium carbonate (CC) 4.0 gr/day). Surgical biopsy showed enlarged hypercellular parathyroid glands, such as diffuse hyperplasia.

She had a satisfactory outcome, with significant regression of extraosseous calcifications 3 months after surgery. The HBD persisted, requiring calcitriol (0.5 *µ*g po/day) and a high dose of CC (6 gr po/day). Despite this postsurgical complication, her bone pain and functional limitations almost disappeared, and the soft-tissue and vascular calcifications diminished after an impressively short time (Figures 1 and 2). The patient could walk without pain and did not need orthosis assistance.

## 3. Discussion

We showed multiple soft-tissue calcifications with high-turnover bone mineral disorder in a PD patient. Despite the increased number of PD patients, most studies related to vascular calcification development have been performed on HD populations, with less attention given to PD patients.

The biochemical pattern found in this patient is striking, with high PTH, but with an AP that is not as high as expected, and increased Ca *x* P product at phosphate expenses (always with mild hypocalcemia). The biochemical pattern in CKD-MBD is vastly heterogeneous, and it is known as the reason for the difficulty to extrapolate these changes to what is happening on the bone; more studies are needed for better conclusions. However, Chen et al. showed that low PTH patterns with high AP tend to occur in patients with PD and are associated with a significant increase in cardiovascular risk [12]. Other groups have revealed an association between AP level and mortality in PD patients [13–15]. This association was not found with high PTH with low or borderline AP, as occurred in our patient. It is noteworthy that most studies report data based on bone alkaline phosphatase and not on total AP [16], as we did in our patient, a weak point in our observations. However, this biochemical pattern of high turnover, with persistent low calcium, could be due to a possible mineralization disorder, where we could find great osteogenic cellularity with dysfunctional osteoblasts in a biopsy [17, 18]. Nevertheless, many authors have found that there is no cutoff point for serum PTH and AP that can predict turnover and mineralization with certainty in both children and adults.

It is important to remember that the multifactorial desensitization of the PTH receptor may explain the low-turnover bone disease as a very prevalent pattern in contemporary CKD patients [19]. Additionally, it can explain the persistent hypocalcemia in our case and even has a close relationship with VC development. It is likely that PTH metabolism dysfunction, with the addition of end-organ hyporesponsiveness, could be the cause of the heterogeneous clinical presentations that we can expect in these patients.

In the case of phosphate at PD, solute removal occurs by diffusion and convection, with an ultrafiltration contribution of approximately 11%. Phosphate clearance is time dependent because in early PD patients, evidence suggests that residual renal function has an important role in phosphate control, with differences between membrane transport characteristics. Nonetheless, over time, the removal trends are lower than those in HD patients [20].

Studies have been shown that PD is associated with low-turnover bone disease, presenting higher phosphate values and more vascular calcification (VC) [21]. De Oliveira et al. showed VC in 77% of 101 patients. It is suggested that morphological changes in the peritoneum induced by long-term PD play an important role in the lower removal of these solutes [21, 22] and could help to develop VC. However, in our case report, we found high-turnover bone disease, with PTHi >1000 pg/ml, but according to other trial suggestions, it seems that high and low bone turnover can impair the mineral buffering capacity and produce VC. It is feasible that both situations can increase the risk of fracture and cardiovascular events in these patients. These findings suggest the importance of appropriate dialysis therapy selection, considering dialysis type change as a cornerstone to achieve better solute removal, as occurred in our case.

Long time in dialysis was a greater risk factor for developing VC than the dialysis modality. Despite that our patient is a young woman, she has received PD for nine years. Her clinical picture may be explained by the imbalance of positive regulators (such as inflammatory cytokines, reactive oxygen species, advanced glycation end products, Ca, P, and iPTH) and negative regulators (such as klotho, matrix-gla protein, fetuin-A, osteoprotegerin, and osteopontin) in the uremic environment [23, 24]. For these reasons, the systematic evaluation of risk factors for VC must be substantial, using tools for early detection, such as hand and pelvic X-rays to produce an Adragao score [9], allowing an early therapeutic strategy. We used this score, although it is not suggested in the guidelines, because of its usefulness and practicality in the detection of peripheral arterial calcification.

The treatment of soft-tissue calcification includes several approaches based on clinical cases, suggesting different strategies because evidence is now lacking. They all focus on aggressive treatment of hyperphosphatemia and avoidance of calcium loading, which seems to be the best management to arrest progress. Thus, discontinuing calcium-containing phosphate binders and synthetic analogs of vitamin *D* and the use of low-calcium dialysate are essential in the treatment of extraosseous calcification [2–4].

The EVOLVE trial showed that a cinacalcet-based therapeutic regimen in hemodialysis patients with advanced secondary hyperparathyroidism (sHPT) reduced VC incidence by 69%–75% [25]. The recent discovery of CaSR in the vascular wall represents a direct effect of calcimimetics on the vasculature leading to VC. Activation of CaSR affects the balance between favor calcification and antagonist calcification molecules. In this way, experimental studies have shown an increase in matrix-gla protein expression in the vascular walls of rats treated with calcimimetics [26]. Despite the rise in calcimimetic use in HD patients, their role in PD patients with sHPT has not shown satisfactory effects in preventing such VC, as recently showed Chow et al. Their study proved the use of cinacalcet in a small group of PD patients and did not improve vascular arterial stiffness using carotid-femoral pulse wave velocity as a surrogate marker. Nevertheless, a reduction of 60.6% in iPTH levels was achieved after one year of treatment [27]. Obviously, more evidence is needed at this point.

Other pharmacological alternatives are being studied for the management of vascular calcification. Recently, Raggi et al. used intravenous myo-inositol hexaphosphate (SNF472) to selectively inhibit the formation and growth of hydroxyapatite in patients with VC at HD [28, 29], achieving calcification score reduction. It seems to be a promising alternative, waiting for new studies to define the impact on the rest of the spectrum of CKD-MBD, such as PTH control, effects on bone remodeling, and soft calcification regression.

In our patient, the concurrence of vascular involvement and high bone turnover refractory to pharmacological measures made surgery the most effective alternative, despite the possibility of late regression of vascular lesions, usually more than 12 months [30, 31]. In general, this alternative is associated with improvements in biochemical parameters of mineral and bone metabolism, cardiovascular health, and mortality [32]. Although, surgery is also associated with serious complications, such as increased risk of hypocalcemia and a 39% increased risk in subsequent hospitalization and postoperative cardiac events, the evidence at this point is contradictory, as some authors have reported better survival and evolution of VC [33, 34] after surgery. In our case, surgical management was decided due to the calcification disease severity and its unresponsiveness to medical management despite the risks, achieving an improvement in biochemical parameters, regression of calcifications, and better quality of life (including less pain and a better gait). The short time after surgery does not allow us to evaluate survival.

PD was a major risk factor for VC in our case, regardless of 9 years in renal replacement therapy. For these reasons, it is important to select properly therapies and look for VC in a routine follow-up using simple, inexpensive, and available tools at any center, especially X-rays. An easy prevention strategy, such as the use of noncalcium phosphate binders, low-calcium dialysis concentrates, nutritional phosphate restriction, and early treatment initiation, is standard management, reserving surgery for complicated cases. Early diagnosis and definitive treatment are crucial to minimize morbidity and mortality.
